# Diabetic Striatopathy: A Rare Movement Disorder Associated With Hyperglycemia

**DOI:** 10.7759/cureus.107250

**Published:** 2026-04-17

**Authors:** Diana J Chang, Kyrstin Lane, Jeffrey Wei, Noriko Salamon

**Affiliations:** 1 Endocrinology, Diabetes, and Metabolism, University of California Los Angeles David Geffen School of Medicine, Los Angeles, USA; 2 Radiology, University of California Los Angeles David Geffen School of Medicine, Los Angeles, USA

**Keywords:** diabetes type 2, diabetic striatopathy, hemichorea associated with non-ketotic hyperglycemia, hemichorea-hemiballismus, hyperglycemia

## Abstract

Diabetic striatopathy (DS), also known as nonketotic hyperglycemic hemichorea-hemiballism, is a rare disorder associated with poorly controlled diabetes mellitus. Presentation typically involves hyperkinetic movement disorders such as hemichorea and hemiballism. It is most commonly seen in females and older adult populations. Associated neuroimaging findings involve hyperintensity on T1-weighted magnetic resonance imaging (MRI) in the striatum and hyperdensity on non-contrast computed tomography (CT). We present a case of an older woman with poorly controlled type 2 diabetes who presents with new hemichoreiform movements in the setting of hyperglycemia that highlights the clinical presentation, diagnostic evaluation, and treatment modalities in managing this rare condition.

## Introduction

Neurologic complications are frequently seen in uncontrolled diabetes, commonly manifesting as peripheral autonomic neuropathy. Diabetic striatopathy (DS) is a rare central nervous system complication of poorly controlled diabetes that presents as hemichorea-hemiballism in the setting of hyperglycemia. It was first documented by Bedwell in 1960 and described by various nomenclatures in the scientific literature, including "hyperglycemic non-ketotic hemichorea/hemiballism", "hemichorea associated with non-ketotic hyperglycemia", and "diabetic hemiballism/hemichorea" [1-3]. The prevalence has been reported to be 1 in 100,000, although there is concern that this value may be underestimated as physicians are often not familiar with this disease process [4]. Cases of DS have been documented throughout the world. However, more than 70% of cases were in Asian countries [5]. DS has been reported across all age groups, but occurs most often in the sixth to seventh decade (earlier in studies done in India) [5, 6]. It is more commonly seen in females with a male-to-female ratio of 1:1.7 [5]. Average blood glucose levels are around 414 mg/dL, and the mean A1c level reported is 13.1% [5]. In addition, most patients are negative for ketone bodies on serum testing (up to 81.7%) [5].

Patients can present with a sudden onset of hemichorea (rapid, involuntary, rhythmic movements affecting unilateral limbs, face, or trunk) or with hemiballismus (more severe, involuntary motions with larger amplitude compared to chorea). Both are hyperkinetic movement disorders often caused by basal ganglia dysfunction [7]. Other reported movement disorders include transient dyskinesia, facial hemispasm, and muscular dystonia [8]. Oftentimes, unilateral symptoms are associated with contralateral basal ganglia changes on imaging [5]. Typical imaging findings include striatal hyperdensity on non-contrast CT or striatal hyperintensity on T1-weighted MRI. In a minority of cases, there are positive neuroimaging findings in asymptomatic patients or symptomatic patients with absent neuroimaging findings. Although several mechanisms to explain the association between hyperglycemia and movement disorders have been proposed, the exact mechanism is unknown. 

This case study examines an elderly Asian woman with poorly controlled type 2 diabetes who presented to the hospital with new-onset hemichorea movements. Our patient's presentation was most consistent with non-ketotic hyperglycemic hemichorea-hemiballism. Our case contributes to the literature by highlighting the rare presentation of neurologic symptoms despite no typical MRI findings. 

## Case presentation

A 96-year-old Asian woman presented to the Ronald Reagan UCLA Medical Center Emergency Department with lethargy and worsening fatigue over the past week. She had a history of hypertension, hyperlipidemia, and poorly controlled type 2 diabetes. Patient reported she had not been checking her blood glucose levels at home for many years. She also had a history of subdural hematoma status post evacuation over 10 years ago. Home medications included benazepril 20mg daily, simvastatin 20mg daily, metformin 1000mg twice daily, and glipizide 5mg daily. Vital signs on presentation were temperature 36.1°C, blood pressure 159/74 mm Hg, pulse 89/min, and respiratory rate 16/min, oxygen saturation 97% on room air. Physical examination revealed an elderly woman, alert and oriented to person, place, and time. Cardiovascular, pulmonary, and abdominal exams were normal. On neurological exam, cranial nerves were intact, muscle strength 5/5 in all extremities with normal reflexes, and intact sensation to light touch. Labs included glucose 730 mg/dL, sodium 126 mmol/L (corrected sodium for hyperglycemia 136 mmol/L), bicarbonate 22 mmol/L, chloride 91 mmol/L, anion gap of 13 mEq/L. Venous blood gas showed pH 7.40 and pCO2 41 mmHg. Beta-hydroxybutyrate was elevated at 8.0 mg/dL (normal range <3.0 ng/dL). Hemoglobin A1c was 17.6% (Table 1). Urine analysis was positive for trace ketones, leukocyte esterase, and nitrites. Urine culture was positive for >100,000 CFU/mL *Klebsiella pneumoniae*.

**Table 1:** Initial test results

Test	Result	Units	Reference range	Interpretation
Sodium	126	mmol/L	135-145	Low
Chloride	91	mmol/L	96-106	Low
Carbon dioxide (CO₂, serum bicarbonate)	22	mmol/L	22-29	Normal
Anion gap	13	mEq/L	8-16	Normal
Glucose	730	mg/dL	70-99	High
pH (venous blood gas)	7.40		7.31-7.43	Normal
pCO₂ (venous blood gas)	41	mmHg	40-60	Normal
Beta-hydroxybutyrate	8.0	mg/dL	<3.0	High
Hemoglobin A1c	17.6	%	<5.7	High

The patient was admitted for three days for urinary tract infection and hyperosmolar hyperglycemic state (HHS). She received IV fluids, antibiotics, and insulin therapy. Blood sugars improved to normal ranges. She was discharged on insulin glargine 5 units at bedtime, metformin 500 mg daily, sitagliptin 25 mg daily for her diabetes management, as well as oral antibiotics.

Two days after discharge, the patient presented again to the emergency department with new involuntary right arm and lip-smacking movements that started after she was discharged home. She denied any numbness, weakness, or episodes of loss of consciousness. She denied any change in cognition or speech. Vital signs were stable. On her neurological examination, the patient was awake, alert, oriented to person, place, time, and situation. Speech was spontaneous and fluent. Cranial nerves were intact. Motor examination showed no pronator drift, good tone with 5/5 strength in all extremities, and normal reflexes. She had notably high amplitude, involuntary choreiform movements of her right upper extremity. She also exhibited bilateral facial dyskinesias that involved the head and neck. No dyskinesia was noted on her left upper extremity and bilateral lower extremities. The patient was admitted to the neurology service. Labs included glucose 242 mg/dL and estimated glomerular filtration rate (eGFR) 79. Sodium, chloride, anion gap, WBC count, troponin I, C-reactive protein, rheumatoid factor, folate, and ceruloplasmin levels were within normal limits (Table 2). RPR was nonreactive, MTB-Quantiferon was negative, and West Nile virus polymerase chain reaction (PCR) testing was negative. Homocysteine, vitamin B1, and copper levels were within normal limits (Table 2).

**Table 2:** Subsequent test results eGFR - estimated glomerular filtration rate; PCR - polymerase chain reaction

Test	Result	Units	Reference range	Interpretation
Sodium	141	mmol/L	135-145	Normal
Chloride	106	mmol/L	96-106	Normal
eGFR	79	mL/min/1.73 m²	≥90	Mildly low
Anion gap	11	mEq/L	8-16	Normal
Glucose	242	mg/dL	70-99	High
White blood cell count	6.06	×10³/µL	4.0-10.5	Normal
Troponin I	<0.04	ng/ml	<0.10	Normal
C-reactive protein	0.5	mg/dL	<0.8	Normal
Rheumatoid factor	<10	IU/ml	<25	Normal
Folate	17.4	ng/mL	8.1-30.4	Normal
Ceruloplasmin	31	mg/dL	17-48	Normal
RPR	Nonreactive	N/A	Nonreactive	Normal
MTB-quantiferon	Negative	N/A	Negative	Normal
West Nile virus PCR	Not detected	N/A	Not detected	Normal
Homocysteine	10	mcmol/L	5-14	Normal
Vitamin B1	81	nmol/L	70-180	Normal
Copper levels	114.4	µg/dL	80.0-155.0	Normal

**Figure 1:**
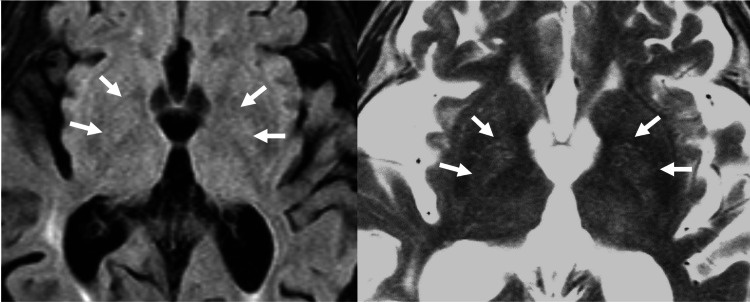
MR brain T1-weighted and T2-weighted MR brain imaging showed no acute infarct, hemorrhage, or intracranial mass effect. There was a bilateral signal abnormality (white arrows) in the globus pallidus. These imaging findings of the basal ganglia are bilateral and non-specific.

EEG showed mild generalized slowing but no epileptiform or other abnormalities that correlated with her movements. MRI and MRA brain imaging showed bilateral high fluid-attenuated inversion recovery (FLAIR) signal in the globus pallidus, which is non-specific (Figure 1).

The presentation was most consistent with non-ketotic hyperglycemia-induced chorea-ballism. The patient was discharged home and had regular follow-up visits with endocrinology for diabetes management. At a post-discharge follow-up appointment with Neurology, the patient and family reported initial resolution of symptoms, but had a recurrence of choreiform movements one month post-discharge. A1c was measured, showing improvement but still high at 12.2%. She was provided counseling on diabetes control and continued follow-up with endocrinology. Subsequent A1c later that year was 7.7%, and the patient reported no recurrence of symptoms at follow-up visits.

## Discussion

Nonketotic hyperglycemic hemichorea-hemiballism (diabetic striatopathy) is defined as the presence of hyperglycemia that is associated with acute-onset choreoballism and/or imaging findings (non-contrast CT demonstrating striatal hyperdensity or T1-weighted MRI showing striatal hyperintensity). It affects 0.05 % of people with diabetes [9]. The majority of patients with DS have type 2 diabetes (96.6% in Chua et al.), and in rare cases, it may present in patients with type 1 diabetes (3.4%) [5]. Nonketotic hyperglycemia, which is now more commonly referred to as hyperosmolar hyperglycemic state, is distinct from diabetic ketoacidosis due to a lack of significant urine ketones. Our patient had only trace ketones, and thus, diabetic ketoacidosis was unlikely here. DS can occur even after hyperglycemia is addressed [5]. This was seen in our case, where the patient developed DS shortly after her first hospital discharge, despite improvements in hyperglycemia. She did not report episodes of hypoglycemia while at home.

The pathophysiology of DS is unknown, but there have been various hypothesized mechanisms in the literature. One proposed mechanism is that in the setting of severe hyperglycemia, brain cells shift to anaerobic pathways of glucose metabolism, leading to gamma-aminobutyric acid (GABA) use as an energy source, which contributes to metabolic acidosis and basal ganglia dysfunction [10]. In addition, depletion of GABA and acetylcholine leads to disinhibition of the subthalamic nucleus and therefore activates the motor cortex through thalamic projections, leading to hyperkinetic movements [11]. This may also explain why DS is less common in patients with diabetic ketoacidosis, as ketone bodies allow for an adequate supply of GABA so that hyperkinetic movement disorders rarely occur [11]. Other mechanisms include microhemorrhage involving the striatum and the development of micro- and macroangiopathy leading to hypoperfusion and ischemia involving the basal ganglia. Further studies are needed to elucidate the underlying mechanism.

As the most common metabolic etiology of hemichorea is diabetes, it is critical to recognize the typical presentation to avoid delayed diagnosis. Most cases occur when there is a non-ketotic hyperglycemia state. Symptoms can also present in the setting of normoglycemia for patients with diabetes [9]. Typically, individuals present with unilateral symptoms affecting the upper and lower extremities, but other presentations have been reported (symptoms involving the face and trunk; symptoms in a bilateral distribution). In some cases, a new stroke can occur in association with DS (can be concurrent with DS or can occur as DS symptoms resolve). Behavior changes, neuropsychiatric concerns, and cognitive changes have also been shown to occur with DS in case reports.

MRI is the test of choice as it is the most sensitive imaging modality (MRI has 95% sensitivity versus CT, which has 79% sensitivity) and is important for early detection of DS [5]. Importantly, Chua et al. found that in 17.5% of cases, there was a discrepancy between CT and MRI findings, with one modality showing no signs of DS. In 14.6% of cases, there was variability in the portion of the striatum that appeared to be involved on imaging. Chua et al. also found that in 9% of cases, the imaging was inconsistent with clinical presentation (i.e., there was normal imaging or the patient was asymptomatic). The inconsistency rate was highest for CT at 21% compared to 5% for MRI. Another study in India found that 55.9% of cases had symptoms but normal neuroimaging [12]. Similarly, our case did not demonstrate imaging findings consistent with the classic clinical presentation. In these cases where imaging may not be diagnostic, it is important for clinicians to be aware of the classic clinical features and use their clinical judgment to avoid delays in diagnosis.

Treatment involves hydration and insulin to improve glycemic control, which is effective in reversing symptoms in up to 25 to 50% of cases [13]. Some patients need additional anti-chorea medications such as antipsychotics, dopamine-depleting agents, benzodiazepines, anticonvulsants, and serotonin reuptake inhibitors. Haloperidol has been identified as one of the most widely used antipsychotics for this condition [5]. Overall, most individuals recover (75 to 100%). One study showed that 47.5% of individuals had full symptom resolution within one week of treatment, 28.8% had full symptom resolution after one week of treatment, and 23.7% had partial recovery [12]. Individuals with no resolution of imaging findings, those who have persistently uncontrolled glucose, and those who have delayed diagnosis may be at risk for recurrence or lack of symptom resolution [13]. This highlights the importance of timely diagnosis. There have also been reports of more aggressive treatment modalities for patients refractory to medical therapy, such as pallidotomy, ventrolateral thalamotomy, transcranial magnetic stimulation, and internal globus pallidus deep-brain stimulation [3,14]. Additionally, approximately 20% of cases recur, so intermittent monitoring is recommended long-term [13].

## Conclusions

In summary, we present the case of an elderly Asian woman with poorly controlled diabetes whose overall presentation was most consistent with a rare case of nonketotic hyperglycemic hemichorea-hemiballism (diabetic striatopathy). Typical imaging findings include striatal hyperdensity on non-contrast CT or striatal hyperintensity on T1-weighted MRI. Our patient had a classic clinical presentation of DS without clear imaging findings. It is important to recognize that patients with DS can have discordance of radiographic findings and clinical symptoms, highlighting the importance of clinical judgment. Clinicians should maintain a high degree of suspicion when evaluating patients with uncontrolled diabetes and movement disorders. Management of hyperglycemia is critical to minimize the risk of this neurologic complication, and in individuals who have developed DS, treatment includes hydration and insulin, and in some cases anti-chorea medications. Early recognition of DS is important as prognosis is generally favorable with intervention.

## References

[1] Bedwell SF (1960). Some observations on hemiballismus. Neurology.

[2] Carrion DM, Carrion AF (2013). Non-ketotic hyperglycaemia hemichorea-hemiballismus and acute ischaemic stroke. BMJ Case Rep.

[3] Son BC, Choi JG, Ko HC (2017). Globus pallidus internus deep brain stimulation for disabling diabetic hemiballism/hemichorea. Case Rep Neurol Med.

[4] Ondo WG (2011). Hyperglycemic nonketotic states and other metabolic imbalances. Handb Clin Neurol.

[5] Chua CB, Sun CK, Hsu CW, Tai YC, Liang CY, Tsai IT (2020). "Diabetic striatopathy": clinical presentations, controversy, pathogenesis, treatments, and outcomes. Sci Rep.

[6] Prabhu S, Ramya N (2012). Movement disorders and diabetes: a study of South India. Internet J Neurol.

[7] Bhidayasiri R, Truong DD (2004). Chorea and related disorders. Postgrad Med J.

[8] Wang W, Tang X, Feng H, Sun F, Liu L, Rajah GB, Yu F (2020). Clinical manifestation of non-ketotic hyperglycemia chorea: a case report and literature review. Medicine (Baltimore).

[9] Wu X, Fu R, Yuan C (2026). Case series of 46 patients with nonketotic hyperglycemia-associated chorea: a retrospective follow-up study. J Clin Endocrinol Metab.

[10] Trinchillo A, Barchetti F, De Joanna G, Esposito M, Piccirillo G, Miniello S (2024). A case of hemichorea associated with nonketotic hyperglycaemia: a new magnetic resonance spectroscopy (MRS) finding and possible future implications. Clin Physiol Funct Imaging.

[11] Arecco A, Ottaviani S, Boschetti M, Renzetti P, Marinelli L (2024). Diabetic striatopathy: an updated overview of current knowledge and future perspectives. J Endocrinol Invest.

[12] Dubey S, Chatterjee S, Ghosh R (2022). Acute onset movement disorders in diabetes mellitus: a clinical series of 59 patients. Eur J Neurol.

[13] Chatterjee S, Ghosh R, Biswas P (2024). Diabetic striatopathy and other acute onset de novo movement disorders in hyperglycemia. Diabetes Metab Syndr.

[14] Kaseda Y, Yamawaki T, Ikeda J (2013). Amelioration of persistent, non-ketotic hyperglycemia-induced hemichorea by repetitive transcranial magnetic stimulation. Case Rep Neurol.

